# Outstanding Student Research: Li et al on Investigating the Placement of Green Carts to Improve Access to Healthful Foods in Food Deserts

**DOI:** 10.5888/pcd11.140338

**Published:** 2014-09-11

**Authors:** Samuel F. Posner

Each year since 2011 *Preventing Chronic Disease* (*PCD*) has issued a special call for student papers for our Student Research Paper Contest. This year *PCD* received 67 submissions from students throughout the world. We are very excited to recognize Kathleen Li and colleagues as the winners of the 2014 contest for their paper entitled “Evaluation of the Placement of Mobile Fruit and Vegetable Vendors to Alleviate Food Deserts in New York City” (1). Ms Li is currently a medical student at the University of California, San Francisco, School of Medicine, and she conducted the study described in her paper with Dr Carol Horowitz and colleagues at the Icahn School of Medicine at Mount Sinai’s Department of Health Evidence and Policy.

In their paper Ms Li and colleagues described their investigation of how the placement of mobile fruit and vegetable vendors affects access to fresh fruit and vegetables in areas that have been designated food deserts. Evidence suggests that people in neighborhoods with access to supermarkets are more likely to have healthier diets and lower levels of obesity than those in food deserts (2). A substantial investment in promotion of mobile fruit and vegetable vendors in the past several years has been at least partially effective in increasing access in areas that lack retail outlets with healthful selections (3–5). The strategic placement of these vendors has been central to the New York City licensing program. In addition to placement, efforts have been made to facilitate the vendor acceptance of Electronic Benefit Transfer (EBT) cards from the Supplemental Nutrition Assistance Program and other assistance programs.

An important aspect of any public health intervention is determining whether the policy is having the intended effect. The New York City program has resulted in issuance of several permits, suggesting that the Green Cart program is changing the landscape in terms of where healthful foods are available. However, looking only at the number of permits does not fully evaluate the intervention. Li and colleagues report that, although Carts were in the streets, they were not in areas designated as food deserts. They were often on the borders of food deserts and in areas where there was already access to healthful foods. These vendors were often in high-traffic areas, close to subway stops and large employers. From a business perspective it is logical to locate in a setting with sufficient traffic so that the enterprise can be profitable. However, from a public health perspective such placement does not fulfill the intent of the intervention. Areas identified as food deserts before the intervention remain deserts with limited access to healthful foods. Green Carts may increase access to healthful foods to the extent that people who live in food deserts go into these high-traffic areas because the carts are on the streets so that they do not have to go into stores. One of the concerns identified by Li and her colleagues is that people who are not mobile may not travel out of their neighborhoods into these higher traffic areas and thus may still have limited access to healthful foods.

Data on licenses suggest that access to healthful foods has increased in food deserts. Li et al’s geographic analysis suggests that New York City Green Carts are mostly on the edges of food deserts and not in the areas of highest need. The data presented in this article are an important component of an evaluation of the Green Cart program; however, the data do not fully answer the question of whether the program is having the desired effect. At least 4 measures remain to be assessed. First, do people who live in these food deserts have access to Green Carts? Access is only the first step. The second measure is purchasing behavior. Data from investigations have shown that vouchers from the EBT and other assistance programs are being used by consumers at Green Carts (3–5). The third area of measurement is consumption of more healthful foods, that is, whether foods purchased are consumed and whether foods are being prepared in healthful ways. The fourth area of measurement is health status of people purchasing from the mobile vendors, because the ultimate goal of these interventions is to improve the health status of the population.

One of the biggest challenges of public health interventions in the field of chronic disease prevention is measuring the intermediate outcomes as well as the health outcomes. This is especially true when evaluating interventions that focus on systems or environmental change. Measuring the number of Green Cart licenses issued is easy. Determining whether the population at high risk is being served requires more sophisticated geographic analysis. Measures of uptake, consumption, and health outcomes can be obtained from surveys and population surveillance; however, without linking exposure, behavior, and outcome, interventions are limited in their ability to answer the question we need answered, that is, does an intervention change the health of the population? As public health interventions focus more and more on systems and environmental change, evaluations are becoming more complicated and take more time.

In the submissions for this year’s Student Research Paper Contest, many of the papers investigated different aspects of systems or environmental interventions. Li and colleagues presented a very interesting and elegant evaluation of one component of the New York City mobile food vendors intervention. However, just as the number of licenses alone doesn’t tell the story, their investigation does not provide a definitive answer to the question of how the intervention is affecting population health. Full understanding of the effect of systems interventions requires the measurement of many processes and intermediate outcomes as well as health outcomes. These health outcomes often take years to be observed. Rarely is there a simple yes or no answer to the effectiveness of a systems intervention. We need to keep in mind that we are trying to change population health outcomes and that these steps along the way are necessary to measure change in that outcome. However, evaluations alone cannot answer all our questions.

One of the challenges in reviewing the papers in this set of contest submissions was that no one study can fully answer the questions we are asking. Li and colleagues are to be congratulated for their innovative and outstanding work and are encouraged to do more to document meaningful changes in intermediate outcomes of interventions and health outcomes.

The Student Research Paper Contest is a great opportunity to recognize the outstanding work being done by the next generation of public health researchers and their mentors. In addition to the 67 students who submitted manuscripts, hundreds of mentors and colleagues supported these young investigators, and we want to recognize them. Without their contributions *PCD* would not have received the number of high-quality papers that it did. Having nearly 70 papers arrive in one day is no small accomplishment. We want to recognize the hard work of *PCD* staff members Sasha Ruiz and Tawni Wold who worked with the authors to make sure that the papers met submission requirements and that the papers were processed in a timely manner. Members of the *PCD* Editorial Board who helped with the initial review (Drs Barbara Bowman and Rachel Kaufman) and with the multiple rounds of review of the top 15 papers (Drs Ana Diez-Roux, Patrick Remington, Sara L Huston, and Eugene J Lengrich) also contributed countless hours reviewing and commenting on these manuscripts. I especially want to recognize Dr Deesha Patel, our 2013 student winner, who served as a reviewer this year. Congratulations and thank you for making the student contest a success!
